# Unraveling the RNA code: a uridine RNA modification drives glycoRNA biogenesis

**DOI:** 10.1038/s41392-024-02056-z

**Published:** 2024-11-27

**Authors:** Marco Sachse, Konstantinos Stellos

**Affiliations:** 1 Department of Cardiovascular Research, Medical Faculty Mannheim, Heidelberg University, Mannheim, Germany; 2 German Centre for Cardiovascular Research (DZHK), Partner Site Heidelberg/Mannheim, Mannheim, Germany; 3 Helmholtz Institute for Translational AngioCardioScience (HI-TAC), Mannheim, Germany; 4 Department of Medicine, University Medical Centre Mannheim, Heidelberg University, Mannheim, Germany

**Keywords:** Biochemistry, Isolation, separation and purification, Target identification, Metabolic engineering, Innate immunity

In a recent *Cell* study, Xie et al.^1^ introduce a novel mass spectrometry-based methodology for the sensitive detection and enrichment of native sialic acid-containing glycoRNA on cell surfaces. This work represents a significant advancement in the emerging field of glycoRNA biology. The authors linked acp^3^U, a modified uridine which was first described five decades ago, with RNA glycosylation in mammalian cells.

**Fig. 1:**
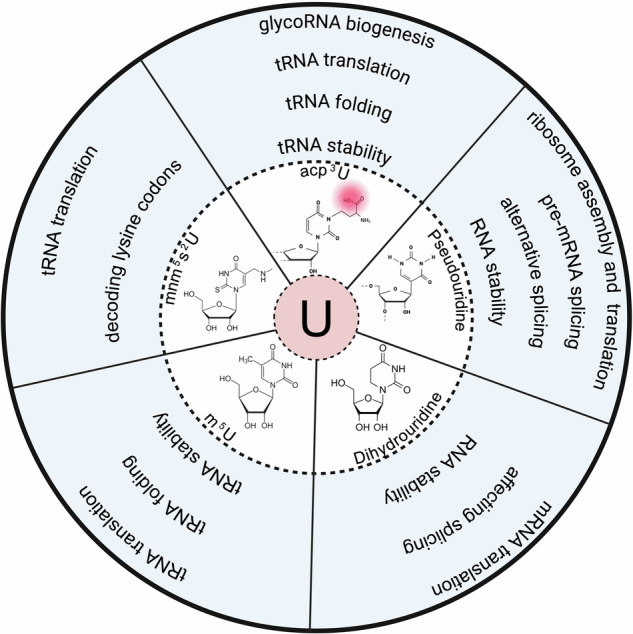
Biological significance of uridine RNA modifications. Pseudouridine regulates pre-messenger RNA (pre-mRNA) splicing, alternative splicing, and RNA stability, influencing ribosome assembly and translation. Dihydrouridine modifications affect RNA splicing and stability, thereby impacting messenger RNA (mRNA) translation. 5-methyluridine (m5U) influences transfer RNA (tRNA) stability, folding, and translation. 5-methylaminomethyl-2-thiouridine (mnm5s2U) supports decoding of lysine codons, impacting tRNA translation. 3-(3-amino-3-carboxypropyl)uridine (acp3U) is important for glycosylated RNA (glycoRNA) biogenesis, stabilization of tRNA structure and tRNA function. Created in BioRender

RNA molecules undergo a variety of post-transcriptional modifications that are essential for their structural integrity, stability, and function, enabling cells to respond to environmental cues. Among these, uridine modifications play critical roles in the life cycle of RNA, influencing processes such as RNA stability, splicing, RNA folding, stabilization of secondary and tertiary RNA structure, translation efficiency and fidelity, and regulation of gene expression (Fig. 1). The discovery of acp^3^U expands the landscape of RNA modifications, revealing that RNA molecules can be directly modified by complex carbohydrates—a phenomenon previously thought exclusive to proteins and lipids. By bridging glycobiology and RNA biology, this finding has the potential to redefine our understanding of cellular communication and function. RNA modifications have long been recognized for their roles in fine-tuning gene expression, influencing RNA stability, localization, and interactions with proteins.^2^ Common modifications include deamination, methylation, pseudouridylation, and acetylation, which are critical for normal cellular processes.^2^ The revelation that entire glycan structures can attach to RNA suggests a new dimension of regulatory potential, indicating that RNAs may participate directly in glycan-mediated cellular interactions. Glycosylation is a critical post-translational modification in proteins, affecting folding, stability, and function. The extension of glycosylation to RNA molecules implies that glycoRNAs may have more dynamic and versatile roles in cellular processes than previously understood, potentially influencing cell-cell communication, immune responses, and disease development.

Prior to this discovery, the existence of glycoRNAs was suggested by studies employing metabolic labeling techniques. Flynn et al.^3^ reported that small noncoding RNAs displayed on the cell surface could be modified with sialic acid-containing N-glycans. Using metabolic labeling with N-azidoacetylmannosamine-tetraacylated (Ac_4_ManNAz), they incorporated azide-modified sialic acids into glycans attached to RNA, enabling their visualization. Despite this previous breakthrough, many questions remain. Metabolic incorporation of modified sugars can be inefficient, leading to sub-stoichiometric labeling and potentially missing a significant portion of glycoRNA. The reliance on metabolic activity meant that only certain cell types could be effectively studied, potentially overlooking glycoRNAs in less metabolically active cells. The labeling targeted the sialic acid residues rather than directly identifying the glycan attachment site on the RNA, leaving the exact nature of the linkage ambiguous.

By addressing some of these questions, the authors developed an RNA-optimized periodate oxidation and aldehyde labeling (rPAL), a method that selectively labels and enriches glycoRNAs. rPAL uses periodate-mediated oxidation of vicinal diols in RNA, converting them into aldehydes that ligate with amine-containing reagents. Compared to the earlier metabolic labeling method using Ac_4_ManNAz, rPAL achieves a 1503-fold increase in signal sensitivity and a 25-fold improvement in signal recovery per RNA mass. This remarkable sensitivity allows the identification of low-abundance glycoRNAs, opening new avenues for studying RNA glycosylation.

Xie et al. validated rPAL using archived RNA samples from HEK293, HUH7, and other cell lines. They detected high-molecular-weight (MW) sialoglycoRNAs, confirmed through enzymatic digestion experiments. Fractionation revealed that the high-MW glycoRNA signal was enriched in membrane fractions but diminished in cytosolic fractions. The rPAL-labeled glycoRNAs were sensitive to live-cell sialidase treatment, with significant signal loss within Vibrio cholerae sialidase treatment, confirming the presence of sialylated glycans on these RNA molecules.

Treating HeLa cells with glycosyltransferase inhibitors P-3FAX-Neu5Ac, NGI-1, and kifunensine, rPAL signal intensity was reduced and altered MW, consistent with earlier findings in Ac_4_ManNAz-labeled sialoglycoRNAs. These results suggest glycoRNA formation is regulated by glycosyltransferases and related enzymes.

To further investigate RNA glycosylation chemistry, Xie et al. employed large-scale RNA-optimized rPAL combined with biochemical purification and SWATH-MS analysis, identifying 34 unique nucleosides from HEK293 and K562 cells. Heavy and light water labeling during enzymatic digestion revealed a mass shift in the acp^3^U signal, confirming the conjugation of glycan moieties. Additionally, synthesized acp^3^U standards exhibited retention times and fragmentation patterns consistent with those of cell-derived acp^3^U in mass spectrometry, validating acp^3^U as a glycosylation site.

Further characterization demonstrated that acp^3^U serves as a template for N-glycosylation. Treatment with PNGase F successfully released glycosylated acp^3^U from RNA leaving COOH residues, indicating that this modified nucleotide is a direct target of glycosylation. Although PNGase F treatment did not significantly diminish overall rPAL signal intensity, it resulted in a substantial molecular weight (MW) shift, thereby reinforcing the role of acp^3^U in N-glycan attachment.

Importantly, the authors further explored the involvement of DTWD2, an enzyme critical for the installation of acp^3^U, by generating DTWD2 knockout clones in U2OS cells. SWATH-MS analysis of these knockout cells revealed decreased levels of acp^3^U and dihydrouridine (acp^3^D), accompanied by reduced rPAL signal intensity. These findings underscore the essential role of DTWD2 in acp^3^U RNA modification and, subsequently, tRNA glycosylation.

Despite these advancements, numerous challenges remain. Mechanistic studies of glycoRNA are necessary to elucidate the enzymatic pathways responsible for the attachment of glycans to acp^3^U, as well as the regulatory mechanisms governing RNA glycosylation across diverse cellular conditions and in response to environmental stimuli. Understanding the molecular determinants of glycoRNA biogenesis will shed light on the functional implications of glycoRNA biology for cell and systems biology and medicine. In a previous study, the authors suggested that glycoRNA on neutrophils might interact with endothelial P-selectin, an adhesion molecule essential for immune cell rolling and adhesion on the vascular wall.^4^ This interaction could potentially aid in neutrophil rolling onto inflamed vascular endothelium.^4^ However, it remains unclear and warrants further investigation whether this finding is directly due to glycoRNA itself or could be attributed to non-specific effects of RNase digestion. Utilizing more sensitive and specific techniques for detecting glycoRNAs—including high-resolution mass spectrometry and advanced imaging methods—while expanding detection methods to identify various glycan structures linked to RNA will enable the mapping of glycoRNAome in transcriptome and its functional role in cell signaling and function. Advancements in detection methodologies for investigating glycoRNA in a cell-specific manner, such as single-cell spatial transcriptomics and RNA in situ hybridization-mediated proximity ligation assays, are emerging. These high-sensitivity tools enable detailed visualization of glycoRNA interactions within tissues, thereby facilitating the mapping of glycoRNA expression and function in various pathological contexts.

Xie et al.‘s work represents a significant step forward in the field of glycoRNA research, providing new tools and insights into the mechanisms of RNA glycosylation. As the field of glycoRNA biology continues to expand, future studies are warranted to uncover new roles for these molecules in health and disease.
